# Consumer Willingness to Pay for Dengue Vaccine (CYD-TDV, Dengvaxia^®^) in Brazil; Implications for Future Pricing Considerations

**DOI:** 10.3389/fphar.2017.00041

**Published:** 2017-02-02

**Authors:** Isabella P. Godói, André S. Santos, Edna A. Reis, Livia L. P. Lemos, Cristina M. R. Brandão, Juliana Alvares, Francisco A. Acurcio, Brian Godman, Augusto A. Guerra Júnior

**Affiliations:** ^1^Programa de Pós-graduação em Medicamentos e Assistência Farmacêutica, Faculdade de Farmácia, Universidade Federal de Minas GeraisBelo Horizonte, Brazil; ^2^SUS Collaborating Centre for Technology Assessment and Excellence in Health, Faculdade de Farmácia, Universidade Federal de Minas GeraisBelo Horizonte, Brazil; ^3^Department of Statistics, Exact Sciences Institute, Universidade Federal de Minas GeraisBelo Horizonte, Brazil; ^4^Programa de Pós-graduação em Saúde Pública, Faculdade de Medicina, Universidade Federal de Minas GeraisBelo Horizonte, Brazil; ^5^Strathclyde Institute of Pharmacy and Biomedical Sciences, Strathclyde UniversityGlasgow, UK; ^6^Division of Clinical Pharmaclogy, Karolinska Institutet, Karolinska University HospitalStockholm, Sweden

**Keywords:** dengue, willingness to pay, vaccine, consumers, Brazil

## Abstract

**Introduction and Objective:** Dengue virus is a serious global health problem with an estimated 3.97 billion people at risk for infection worldwide. In December 2015, the first vaccine (CYD-TDV) for dengue prevention was approved in Brazil, developed by Sanofi Pasteur. However, given that the vaccine will potentially be paid via the public health system, information is need regarding consumers’ willingness to pay for the dengue vaccine in the country as well as discussions related to the possible inclusion of this vaccine into the public health system. This was the objective of this research.

**Methods**: We conducted a cross-sectional study with residents of Greater Belo Horizonte, Minas Gerais, about their willingness to pay for the CYD-TDV vaccine.

**Results**: 507 individuals were interviewed. These were mostly female (62.4%) had completed high school (62.17%), were working (74.4%), had private health insurance (64.5%) and did not have dengue (67.4%). The maximum median value of consumers’ willingness to pay for CYD-TDV vaccine is US$33.61 (120.00BRL) for the complete schedule and US$11.20 (40.00BRL) per dose. At the price determined by the Brazil’s regulatory chamber of pharmaceutical products market for the commercialization of Dengvaxia^®^ for three doses, only 17% of the population expressed willingness to pay for this vaccine.

**Conclusion**: Brazil is currently one of the largest markets for dengue vaccine and the price established is a key issue. We believe the manufacturer should asses the possibility of lower prices to reach a larger audience among the Brazilian population.

## Introduction

Dengue is an arbovirosis transmitted to humans by the bite of a mosquito of the *Aedes* genus, especially, *Aedes aegypti*. It is estimated that 390 million infections occur annually worldwide (Gubler, 2011; Bhatt et al., 2013), with the number of cases of dengue increasing in frequency and geographic region (Guzman et al., 2010; Brady et al., 2012; Simmons et al., 2012; Bhatt et al., 2013). Based on mathematical modeling, the global annual incidence has been estimated at approximately 50 to 100 million symptomatic cases each year in recent years (Beatty et al., 2011; Bhatt et al., 2013). This flavivirus represents an important social and economic impact in most tropical and subtropical countries, and it is currently estimated that approximately US$5million are spent annually on hospitalizations related to dengue worldwide (Suaya et al., 2009). However, this is now likely to be a considerable under-estimate.

Dengue is endemic in Brazil, with a high and increasing incidence in recent years (Brasil, 2011, 2013, 2016). In 2015, there were 1,587,080 registered cases probably due to dengue, 839 deaths and an incidence of 782.6 cases /100,000 inhabitants. In the State of Minas Gerais, there was a demonstrated incidence of 879.8 cases/100,000 inhabitants and 67 deaths (Brasil, 2016). Data released by the National Information System of Notifiable Diseases (SINAN) demonstrated the occurrence of 2,320,956 and 4,406,767 cases of dengue in the state of Minas Gerais and Brazil respectively between 2010 to 2014 (Brasil, 2014a). In 2014, more than 150 million Brazilian reais (US$42.016million) were spent on surveillance, prevention and control of dengue and chikungunya virus in Brazil (Brasil, 2014b).

Until now, there has not been a specific licensed treatment for dengue, and the development of effective vaccines against all four serotypes of DENV is an important strategy to control this flavivirus and significantly contribute to reducing the disease burden (Webster et al., 2009; Durbin and Whitehead, 2011; McArthur et al., 2013). Common strategies to help control dengue include preventing mosquitoes from accessing egg-larving habitats, using environmental management interventions such as removing artificial man-made mosquito habitats, emptying and cleaning domestic water storage containers, as well as personal and household protection including applying insecticides (Brasil, 2002; World Health Organization [WHO], 2016).

Recently, the tetravalent chimeric vaccine CYD-TDV from Sanofi Pasteur was approved for the prevention of dengue in endemic countries including Mexico, the Philippines, El Salvador, Costa Rica, Paraguay and Brazil (Brasil, 2015a,b; Dengue Vaccine Initiative [DVI], 2015; Roland and Bisserbe, 2015). The disappointing results in individuals under 9 years of age (Hadinegoro et al., 2015) led to vaccine being indicated for the population 9 years or older. The resultant approved indication from this first dengue vaccine is for individuals 9–45 years (e.g., Brazil) or 9–60 years of age (e.g., Paraguay), depending on the license (World Health Organization [WHO], 2016).

CYD-TDV was evaluated during the active phase of surveillance (25 months post-enrolment) in CYD14 (Capeding et al., 2014) and CYD15 (Villar et al., 2015). As per the protocol, vaccine efficacy against virologically confirmed symptomatic dengue illness was 56.5% (95% CI; 43.8% – 66.4%) in CYD14, assessed in Asia, and CYD15 with 60.8% (95% CI; 52% – 68%) evaluated in clinical trials conducted in Latin America including Brazil. Sanofi Pasteur recommended the administration of three doses each 6 months apart (World Health Organization [WHO], 2016). However, the complete duration of vaccine protection is still unknown (Hadinegoro et al., 2015; World Health Organization [WHO], 2016).

The Brazil’s regulatory chamber of the pharmaceutical products market (CMED) is responsible for evaluating and establishing the prices of medicines for commercialization in Brazil by Resolution n°2 of 5 March 2004, referencing prices for the same medicines in other countries including Australia, Canada, and the United States (Brasil, 2004). From prices established by CMED, pharmaceutical companies may apply for incorporation of their products into the national health system by sending a process submission to the National Commission on Technology Incorporation of the National Health System (CONITEC) (Brasil, 2008). In this context, endemic countries, including Brazil, will have to make important decisions such as the possible incorporation of this vaccine into their public systems within a context of constrained budgets. In this scenario, pharmacoeconomic evaluations, such as the assessment of willingness-to-pay and cost-effectiveness analysis, are important for decision-making (Palanca-Tan, 2008; Lee et al., 2015).

Willingness-to-pay (WTP) is a relevant methodological approach to estimate the maximum amount that an individual is willing to allocate to programs, services and health technologies. It is usually applied in cost-benefit analysis and in health technology assessment (Haab and Mcconnell, 2002). The lack of available WTP studies with the Brazilian consumer for a dengue vaccine, and the possible upcoming vaccination with CYD-TDV in the country, is a concern given the potential budget impact and the current economy situation. This study sought to estimate the Brazilian consumers’ willingness to pay for this vaccine in order to contribute to the debate and pharmacoeconomic reviews focusing on demand and potential prices for dengue treatments in Brazil.

## Materials and Methods

This study estimated the willingness to pay of Brazilian consumers toward the CYD-TDV dengue vaccine through an analysis of contingent valuation, which enables evaluation of the monetary amount an individual is willing to pay to acquire a certain product or service using questionnaires with direct questions. The respondents did not have the disease at the time of the interview, but they may or may not have had dengue in the past (Haab and Mcconnell, 2002).

### Design and Study Location

The survey was conducted in the metropolitan region of Belo Horizonte, capital of Minas Gerais State, the second most populous state in Brazil. Minas Gerais state has 21,013,869 inhabitants with 2,375,151 inhabitants currently registered in the Belo Horizonte metropolitan region. In addition, Belo Horizonte and Brazil presented, respectively, a mean Human Developed Index of 0.810 and 0.737 in 2010 (Atlas do Desenvolvimento Humano no Brasil, 2016).

Minas Gerais State is similar to Brazil as a whole for certain aspects including mean income per capita and socio demographics. Mean income per capita was US$315.97 (1128.00BRL) for Brazil and US$311.76 (1113.00BRL) per month for Minas Gerais in 2015 (Instituto Brasileiro de Geografia e Estatística [IBGE], 2014; Agência Brasil, 2016). In addition, despite that there being 26 States in the country, Minas Gerais has one sixth of the Brazilian cities and represents a relevant epidemiological context for the flavivirus (Brasil, 2014a, 2016). In addition in 2013, Minas Gerais was the State with the highest number of dengue cases in the country (Brasil, 2014a). Consequently, providing a robust sample for the study.

Participants were interviewed using a questionnaire developed by the research team, based on a literature review (Haab and Mcconnell, 2002; Palanca-Tan, 2008; Lee et al., 2015). The survey was conducted in May 2016 and the interviewers were undertaken by graduate and undergraduate students of the School of Pharmacy of the Federal University of Minas Gerais, trained to conduct interviews and answer possible questions.

### Data Collection Instrument

The technique for measuring the willingness to pay is the application of a questionnaire, with prior presentation to the respondent of all the features of the disease and the intervention necessary for decision-making, as well as the involved conditions and important aspects of the clinical context of the disease. To fully implement this technique, it is essential that all participants have received the same information. This was assured by specific and intensive training of the interviewers (Haab and Mcconnell, 2002).

The questionnaire was divided in five sections: (1) Questions to understand what the participants knew about dengue; (2) Information about the disease, intervention and alternatives for disease prevention; (3) Questions to test the understanding of the information provided; and (4) Discrete Choice, Bidding Game and Open-Ended questions (Haab and Mcconnell, 2002). Section 5 consisted of a self-reported socioeconomic questionnaire. The questions in section (4) were included in order to assess whether individuals would be willing to pay US$54.05 (180.00 BRL) for the three-dose scheme of CYD-TDV vaccine as well as obtain an estimate of a range of values and a point estimate that respondents would pay for the technology.

US$54.05 for three doses of CYD-TDV for the Discrete Choice technique was established based on the maximum price for the consumer of the yellow fever vaccine – US$19.50 (64.92BRL) (Fiocruz Laboratory), established by CMED in 20 July 2016 (Agência Nacional de Vigilância Sanitária [ANVISA], 2016).

All questions related to the research context and/or difficulties in completing the socioeconomic questionnaire were clarified by the interviewers. The information about the mean effective protection for all four serotypes (∼60%), as well as the possibility of local (e.g., swelling at the site of application and pain) and systemic (e.g., fever, myalgia, asthenia, and headache) adverse events were included in the text read to all participants in the initial stage of the interview. There was also a figure explaining graphically the efficacy of the vaccine to aid the dissemination of information.

### Sampling and Selection Criteria

Interview selection was random. Passers by in major circulation paths, close to parks, markets and fairs in the metropolitan region of Belo Horizonte were invited to participate and, if they agreed, answered the questionnaire in the same location. Considering the scenario with higher uncertainty that is 50% respondents agreeing to pay the value of US$54.05 (180.00BRL), with a two-sided 0.05 significance, we calculated a minimum of 400 respondents would be required in this research.

Individuals could or not have history of dengue, but could not presently have symptoms or have a diagnosis of the disease at the time of the interview. To be selected, individuals must have declared that they have an income. Individuals under 18 without their own income were excluded. In addition, participants who showed willingness to pay higher than twice the value of their declared monthly income and individuals who would not use this vaccine, even if it would be free, were excluded from the analysis in line with previous publications (Lee et al., 2015).

### Data Analysis

The willingness to pay for dengue vaccine was estimated by the median of the maximum declared value by the individuals who were willing to pay any amount greater than or equal to zero. The median among groups defined by covariates were compared using the Mann-Whitney test (two groups) or Kruskal–Wallis test (three or more groups). The significance level was 5%. All socioeconomic variables were evaluated and the relation with the maximum value of willingness to pay for CYD-TDV, such as education and income were included. To assess income variation, we stratified the value of “<3”; “3–10” and “>10” times the minimum wage, in order to measure the percentage of individuals for each range.

Furthermore, we measured the frequency of the participants that have or not private health insurance. According to the National Regulatory Agency for Private Health Insurance and Plans (ANS), that regulates the private health insurances in Brazil, Minas Gerais has coverage of private health insurance of between 20 and 30%, with 5,467,559 beneficiaries in the State in 2014. The coverage in Brazil was 25.2% of the population (48,824,150 individuals) in March 2016 (Agência Nacional de Saúde Suplementar [ANS], 2016a,b).

In addition, we further evaluated the relation of willingness to pay by individuals that had previously had or not dengue. Statistical analysis was performed using Microsoft Excel 2007, R (R Core Team, 2014) and Minitab 17. For comparison purposes, we adopted the conversion value established by the World Bank for Purchasing Power Parities (PPPs) (2015: 1 US$1 = 3.330BRL).

### Ethics Statement

All interviews were conducted after reading and signing the Term of Free and Clarified Consent. All researchers of the project signed a confidentiality agreement prior to the interviews. This study was approved by the Ethics Committee of the Federal University of Minas Gerais (COEP) under the CAAE 57219816.0.0000.5149.

## Results

### Population Characteristics

We conducted 507 interviews with individuals aged between 18 and 84 years old who agreed to participate and answer the questionnaire. The mean age of respondents was 34.6 ± 12.8 years, 37.6% were male, 74.4% were working at the time of interview, and 37.8% had completed higher education (**Table 1**).

**Table 1:** Characteristics of the respondents.

Variable	*n*	(%)^∗∗^
Age in years [mean (SD)]	34.6	12.5%
Men	188	37.6%
Has children	197	(38.9%)
Educational level		
Had never attended school	2	0.4%
Complete primary education	46	9.3%
Completed high school	261	52.5%
Complete college or more	188	37.8%
Currently working	372	74.4%
Have health insurance	318	64.5%
Dengue history	162	32.6%
Had dengue and reported having used only the public health system	68	49.3%
Had dengue and reported having used only a private health provider	54	39.1%
Had dengue and reported have used both services	16	11.6%
Reported that other people in the household had dengue	212	43.8%
**Family income (number of minimal wages)^∗^**		
<1		8.5%
1–2		15.4%
2–3		15.2%
3–5		20.7%
5–10		22.5%
10–20		7.9%
>20		1.2%

*^∗^8.6% of respondents refused to answer on family income. ^∗∗^The value of the difference to reach 100% in all questions, are due to answers such as “I do not know” and “I do not want to answer.”*

The participants who reported a history of dengue were 32.6%, the utilization of public health service was reported by 49.3%, and those respondents who reported at least one dengue case in the household were 43.8%. In approximately 70% of respondents, their family income was below five times the minimum wage (**Table 1**).

### Willingness to Pay for CYD-TDV Dengue Vaccine

Of the 507 subjects, 7.3% said they would not be vaccinated even if CYD-TDV did not have any cost. The main reasons for this were efficacy (37.8%) and safety (40.5%). In addition, only three (8.1%) respondents said they did not use any vaccines and 59 (11.6%) said they would use this vaccine only if it would be provided free of charge. Considering these exclusions criteria, 464 respondents were eligible for the WTP analysis.

**FIGURE 1:**
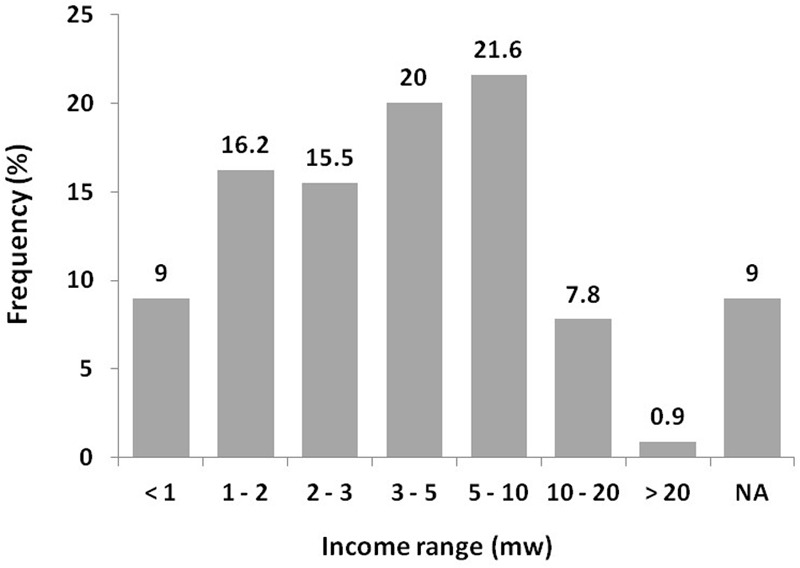
**Family income of respondents included in analysis of the willingness to pay for dengue vaccine (CYD-TDV) in this study.** ^∗^NA, not available – The respondents that answered, “I do not know and I do not want to answer”; mw, minimal wage.

Among these 464 individuals, 37.9% were men, 88.8% had completed high school or more, 39.2% had children, 73.7% were working at the moment of interview, 62.1% had health insurance and 31.7% had previously had dengue. The participants who reported a family income up to 10 times the minimal wage were 83.4% (**Figure 1**).

With the application of the Discrete Choice Technique, it was found that 44% of participants were willing to pay US$54.05 (180.00BRL) for the three-dose regimen of the vaccine. Of the 190 respondents who had children, 131 (68.9%) were willing to pay USD$54.05 for CYD-TDV vaccination of their family. Results of the Bidding Game technique revealed that, in general, the amount the respondents were willing to pay ranged from US$27.03 (90.00BRL) to US$108.11 (360.00BRL), representing 54.1% of individuals involved in interview. The minimum and maximum willingness to pay for three doses of CYD-TDV vaccine were of 0.00 and 1,800.00 BRL.

**FIGURE 2:**
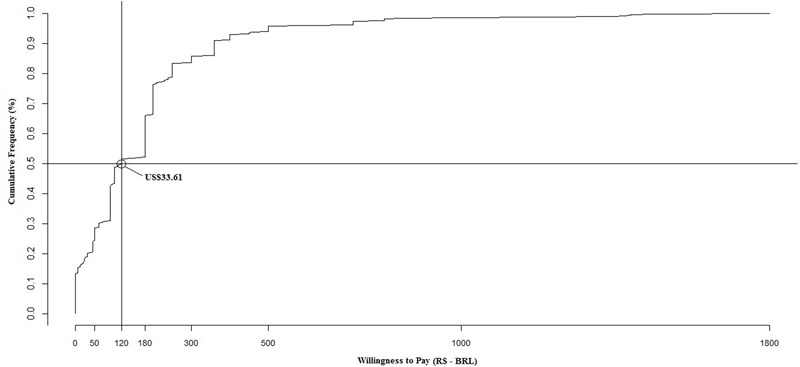
**Cumulative percentage of individuals willing to pay for CYD-TDV vaccine according to the maximum values reported**.

The willingness to pay for dengue vaccine by the Brazilian consumer was estimated at the median value of US$36.04 (120.00BRL) for the three-dose regimen or US$12.01 (40.00BRL) per dose. This means that 50% of individuals interviewed reported maximum values of willingness to pay equal to or less than US$36.04 (**Figure 2**).

**FIGURE 3:**
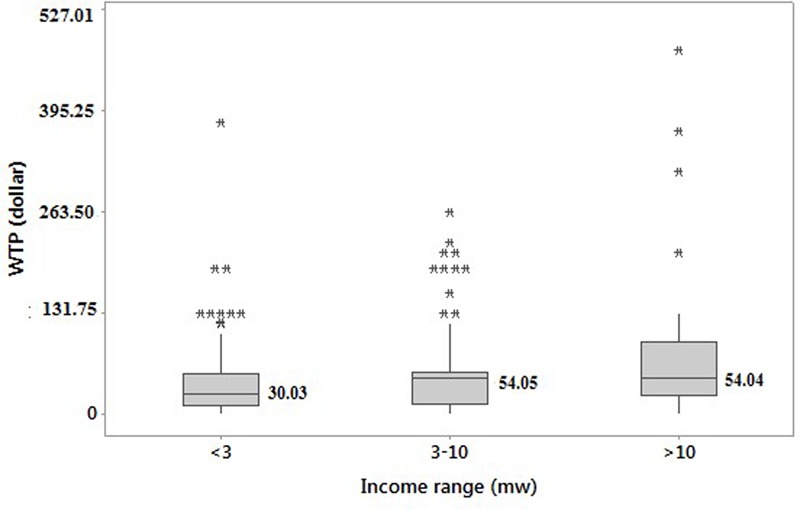
**Box plot of the willingness to pay for three doses of CYD-TDV according to family income.** NB. The results were statistically significant (<0.05); (^∗^) Extreme values presented for each income range; mw, minimal wage.

The only variable correlated with willingness to pay with statistical significance (<0.05) was monthly income (*p* = 0.003) when stratified as “<3,” “3–10” and “>10” times the minimum wage, representing family income values under US$739.50 (2,640.00BRL) (30.8%), between US$792.79 (2,640.00BRL) and US$2,642.64 (8,800.00BRL) (34.1%) and above US$2,642.64 (6.9%), respectively, per month. Median values of willingness to pay for these three groups were respectively US$30.03 (100.00BRL) and US$54.05 (180.00BRL) for the highest income groups (**Figure 3**). As expected, the increase of family income contributed to a higher willingness to pay, which is logical and consistent with the published theory (Haab and Mcconnell, 2002).

## Discussion

This study sought to estimate the willingness to pay among Brazilian consumers for the dengue vaccine recently licensed in the country, with the study population having similarities with the profile of the Brazilian population as a whole. This included certain characteristics such as higher percentage of women (51.4%), a low percentage of individuals who have never studied (8.5%) and the percentage of individuals with at least 11 years of education (41.8%) (Instituto Brasileiro de Geografia e Estatística [IBGE], 2014). The percentage of families in our study with income less than 5 times the minimum wage was just under 70% (**Table 1**). This value was below the Brazilian Institute of Geography and Statistics (IBGE) in their profiling family in the national context, where the percentage was 87.9%. This might contribute for a higher median value of willingness to pay for the vaccine compared to the national population (Instituto Brasileiro de Geografia e Estatística [IBGE], 2014).

In this study, the percentage of respondents who reported having health insurance was 64.5% (Agência Nacional de Saúde Suplementar [ANS], 2016b). The Brazilian private market for health insurance is strictly regulated by the National Regulatory Agency for Private Health Insurance and Plans, which works on behalf of the Ministry of Health. Private health insurance can either be purchased individually or obtained as a work benefit, depending on the employer. The Brazilians that decide to purchase private health insurance may still access public health services if they wish or need. The Brazilian public health system, named SUS, was established in 1988 by constitution in order to guarantee access to health care to the entire population. The public system maintains primary and outpatient centers, hospitals, diagnostic laboratories and should provide access to pharmaceuticals including vaccines (Rizzotto and Campos, 2016).

The median willingness to pay value for CYD-TDV was US$33.61 (120.00/11.20BRL dose) for the three-dose scheme and clinical efficacy of 60% (Hadinegoro et al., 2015; Godói et al., 2016). The reason for expressing values in medians in willingness to pay analysis (Palanca-Tan, 2008; Hadisoemarto and Castro, 2013; Lee et al., 2015) is that the mean is sensitive to outliers, which may contribute to an erroneous perception of what the population is indeed willing to pay. The median value of US$33.61 shows the maximum amount 50% of respondents would be willing to pay; however, this does not represent an estimated average number of people willing to pay for the vaccine (Buckland and Macmillan, 1999).

This study is the first study in Brazil to consider the actual scenario of a possible vaccination with CYD-TDV with clinical information arising from clinical phase III trials (Capeding et al., 2014; Villar et al., 2015) and from the 25 months follow-up study (Hadinegoro et al., 2015). Other studies adopted a hypothetical vaccination scenario with 100% safety and efficacy and with protection for 10 years and for life, as seen, respectively, in the studies conducted in the Philippines (Palanca-Tan, 2008) and Indonesia (Hadisoemarto and Castro, 2013). However, we believe this is an unrealistic scenario given the current clinical information.

The number of doses used in the studies was also variable. A single dose study was conducted in the Philippines (Palanca-Tan, 2008) and Indonesia (Hadisoemarto and Castro, 2013) and three doses in Vietnam, Thailand and Colombia (Lee et al., 2015), which is similar to our study. Among the respondents in our study, 7.3% reported not wishing to be vaccinated even if the vaccine was free of charge. The same situation happened in the studies from Vietnam, Thailand, and Colombia (Lee et al., 2015).

The willingness to pay of Brazilian consumers of US$33.61 (BRL120.00) is closer to that observed in endemic countries such as Vietnam and Colombia. This is between the values found in Vietnam at US$26.13 and the Philippines at US$60.00. The observed values in Indonesia, Colombia, and Thailand and were respectively US$ 1.94, US$22.60 and US$ 69.78. The studies published in the context of willingness to pay for dengue, in general, considered a hypothetical dengue vaccine with results of efficacy, safety and protection time better than the results seen with CYD-TDV in phase III clinical trials (Palanca-Tan, 2008; Hadisoemarto and Castro, 2013; Lee et al., 2015). Consequently, again questioning the findings.

The Brazil’s regulatory chamber of pharmaceutical products market is an inter-ministerial body responsible for price setting. New-patented innovative products such as Dengvaxia^®^ are classified as Class I. As a result, manufacturer prices may not exceed the lowest price in the following markets: Australia, Canada, France, Greece, Italy, New Zealand, Portugal, Spain, or United States of America (Brasil, 2004). However, since CYD-TDV is not marketed in these countries, CMED had no comparison to establish a price for vaccine in Brazil. On July 25th of 2016, CMED reported that the manufacturer price for each Dengvaxia^®^ dose in Brazil may vary from US$37.19 to US$38.80 (132.76–138.53BRL) according to the States (provinces) tax rates of each of the 26 states of Brazil. For Minas Gerais State, the maximum consumer price is US$37.71 (134.63BRL). Considering the need for three doses of the vaccine to achieve planned efficacy, the amount paid for each person vaccinated will be at least US$113.13 (403.89BRL), which represents the willingness to pay of only 17% of the population in this study.

Mahoney et al. (2012) studying the production costs of another dengue vaccine, which is being developed at the Butantan Institute, found that the production scale (15 million doses per year) in ten vials should cost around US$0.51 to US$0.65 per vial. When the quantity produced increases to 60 million doses per year, the cost of production could potentially fall to US$0.20 per dose. The authors demonstrated that vaccines for Japanese encephalitis and type A meningitis are available in developing countries at prices below US$1.00 per dose. This is much lower than consumer prices demanded by manufacturer in Brazil or established by CMED.

Brazil has a comprehensive immunization program with coverage for an appreciable number of infections. In future scenarios, we believe public health systems purchasing the dengue vaccine should assess carefully the cost-effectiveness ratio in combination with a budget impact analysis, as the efficacy of this new dengue vaccine may be considered insufficient compared to other vaccines for similar conditions or disease burden with appreciably lower prices. Comparisons with other vaccines prices and effectiveness for diseases with similar burden may contribute to political decisions regarding the possibility of incorporating this technology into public health systems at acceptable and reasonable prices, bearing in mind the current economic climate in Brazil and the desire to continue to offer universal healthcare. Such discussions have grown in recent times driven by the increasing prices for new medicines especially new cancer medicines and those for orphan diseases, despite costs of research and production estimated at US$50 to 100 million per compound by some authors (Experts in Chronic Myeloid Leukemia, 2013; Phelan and Cook, 2014; Godman et al., 2015; Howard et al., 2015; Tefferi et al., 2015; Bruijn et al., 2016).

In a study conducted in Brazil, Araújo et al. (2016) estimated the potential impact of vaccination against dengue. In a more conservative scenario, the authors estimated a 22% reduction in cases of dengue (routinely vaccinate up to 9 years old and vaccination campaign up to 10 years) and 81% in the liberal scenario (routine to 9 years and vaccination campaign up to 40 years) over 5 years. Furthermore, they demonstrated that vaccination could reduce 233,000 hospitalizations due to the disease during the considered period. CYD-TDV was licensed for individuals with age between 9 and 45 years old (Brasil, 2015a). However, it is important to balance this against the potential budget impact. This especially as it is important to emphasize that all efforts and strategies for vector control by governments and society will need to continue since the vaccine has only 60% efficacy, and especially because of other existing arboviruses, such as Chikungunya and Zika (Tilak et al., 2016), with Zika related to microcephaly epidemics in Brazil (Brasil, 2015b).

The contingent valuation is the most common approach to estimate the monetary value for goods and services from hypothetical questions (Haab and Mcconnell, 2002; Palanca-Tan, 2008; Hadisoemarto and Castro, 2013; Lee et al., 2015). However, some limitations are noted such as the respondents may not have full information (e.g., disease, severity and frequency of symptoms) or that it may simulate a scenario very distinct and different compared with the real situation (e.g., efficacy of the intervention). To avoid such limitations, the questionnaire used must be complete and up to date and include all relevant situations and conditions related to the intervention or service in the analysis to avoid possible bias (Boyle, 2003). This study was conducted using efficacy results extracted from the analysis of 27,355 individuals from 2 to 16 years old (Hadinegoro et al., 2015). These results were used to grant commercial license to Dengvaxia^®^ for children as well as adults in Brazil and in other countries (Godói et al., 2016). Consequently, helping to address such concerns. Real-world results of the vaccine are currently missing, which may overestimate the value of the WTP (if effectiveness is lower), or underestimate the value (if effectiveness is higher – most unlikely scenario). In addition, the respondent’s willingness to pay were constrained within the attributes and levels presented in this study. Lastly, the random sample used may not be fully generalizable to population of Brazil as a whole. However, despite these limitations, we believe our findings do provide guidance to the Brazilian authorities and the manufacturers of the vaccine on potential pricing and reimbursement strategies.

## Conclusion

Despite the limitations regarding income differences between citizens in Minas Gerais and Brazil as a whole as well as limitations with the sampling method, we believe this study provides important information about how much consumers are willing to pay for the CYD-TDV vaccine approved in Brazil to avoid the risk of being infected. From the price determined by CMED for commercialization of Dengvaxia^®^ (Minas Gerais), i.e., US$113.13 for three doses, only 17% of the participants involved in this study were willing to pay for this vaccine, and this from a higher income base than Brazil as a whole. This is a concern given the current resource constrained environment in Brazil. We believe, based on our study findings, that the manufacturers may wish to reconsider their pricing strategy. This is because Brazil constitutes one of the largest markets for dengue vaccine and there are appreciable competing demands on available resources, especially given the current economic situation in Brazil.

## Author Contributions

IG, LL, JA, FA, and AGJ devised the study and the instruments as well as wrote the first draft; IG, AS, LL, ER and CB helped undertake the study and the analysis; IG, LL, JA, FA, BG, and AGJ subsequently revised the draft and produced the final manuscript. All authors approved the final manuscript.

## Conflict of Interest Statement

The authors declare that the research was conducted in the absence of any commercial or financial relationships that could be construed as a potential conflict of interest.
